# Biodiversity and multifunctionality in a microbial community: a novel theoretical approach to quantify functional redundancy

**DOI:** 10.1098/rspb.2013.2498

**Published:** 2014-02-07

**Authors:** Takeshi Miki, Taichi Yokokawa, Kazuaki Matsui

**Affiliations:** 1 Institute of Oceanography, National Taiwan University, Number 1, Section 4 Roosevelt Road, Taipei 10617, Taiwan, Republic of China; 2 Center for Marine Environmental Studies (CMES), Ehime University, 3 Bunkyo-cho, Matsuyama, Ehime 790-8577, Japan; 3 Laboratory of Environmental Biological Science, Faculty of Science and Technology, Kinki University, 3-4-1 Kowakae Higashiosaka, Osaka 577-8502, Japan

**Keywords:** ecosystem function, microbial diversity, multifunctional redundancy, accumulation curve, orthologue richness, species loss

## Abstract

Ecosystems have a limited buffering capacity of multiple ecosystem functions against biodiversity loss (i.e. low multifunctional redundancy). We developed a novel theoretical approach to evaluate multifunctional redundancy in a microbial community using the microbial genome database (MBGD) for comparative analysis. In order to fully implement functional information, we defined orthologue richness in a community, each of which is a functionally conservative evolutionary unit in genomes, as an index of community multifunctionality (MF). We constructed a graph of expected orthologue richness in a community (MF) as a function of species richness (SR), fit the power function to SR (i.e. MF = *c*SR*^a^*), and interpreted the higher exponent *a* as the lower multifunctional redundancy. Through a microcosm experiment, we confirmed that MF defined by orthologue richness could predict the actual multiple functions. We simulated random and non-random community assemblages using full genomic data of 478 prokaryotic species in the MBGD, and determined that the exponent in microbial communities ranged from 0.55 to 0.75. This exponent range provided a quantitative estimate that a 6.6–8.9% loss limit in SR occurred in a microbial community for an MF reduction no greater than 5%, suggesting a non-negligible initial loss effect of microbial diversity on MF.

## 1. Introduction

The rapid and continued development of molecular biology and genomic techniques has unveiled immense soil, freshwater and ocean microbial diversity [1–8]. However, the quantitative relationship between microbial diversity and ecosystem function remains unclear. Recent advances in our understanding of plant diversity and ecosystem function in terrestrial ecosystems, which have been achieved by large-scale field experiments [9–13] clearly demonstrate the paucity of studies in microbial communities. Instead of field experiments that manipulate natural microbial diversity levels, ecologists have applied microbial communities as a ‘model’ to test general theory in microcosm settings (see [14,15] for a review). Even in experiments considering relatively high species richness (SR), results indicated the levels of manipulated microbial richness are less than 100, much lower than levels in natural communities [16]. Furthermore, it remains unclear whether variation in ecosystem functions was attributable to differences in community composition at the species level, highly frequent horizontal functional gene exchange among species [17,18], or rapid functional trait evolution of individual species [19].

Many studies have demonstrated that the relationships between microbial diversity and ecosystem functions are weak [20 and references therein]. Microbial decomposer communities often exhibit high redundancy for a single function, such as microbial respiration and biomass production, which has been shown in more extensive plant biodiversity and ecosystem function studies in terrestrial systems [13,21]. Nielsen *et al.* [20] reviewed 57 studies, and concluded that the saturating relationship between microbial richness and a single ecosystem function was dominant in soil ecosystems, suggesting high functional redundancy in soil microbes. In aquatic ecosystems, linear and saturating patterns are a challenge to distinguish owing to a limited range in microbial richness. The relationship demonstrated by experiments (SR was directly manipulated or indirectly manipulated by a dilution–extinction method) was positive [22–25], negative [24] or non-significant [23–27]. A correlation between microbial richness and a function observed in environmental gradients was positive, fit by an exponential function [28], linearly negative [29] and nonlinearly negative for bacterial production and bacterial respiration [30]. It is notable that the relationship differed when focused on alternative functions [23–25,30]. Some studies indicated it was not SR, but heterogeneity in community composition that explained variability in some functions [24,25,31–36], however, a significant relationship between composition and function was not detected in other studies [32,35,37,38].

Previous studies found that functional redundancy in microbial communities was high [16,20], suggesting an initial loss in microbial diversity was unlikely to substantially affect ecosystem functions. However, this view (i.e. low effects from initial biodiversity loss on ecosystem functions) was highly sensitive to quantitative measures of microbial function. Peter *et al.* [24,27] and Langenheder *et al.* [32] demonstrated that if the focal function was more specific (e.g. ability to decompose recalcitrant carbon substrates) than general functions (e.g. respiration and biomass production), the link between SR or community composition and function was greater. More importantly, multifunctional redundancy was generally lower (the degree of multiple functional dependence on diversity was higher) than single-functional redundancy [27,39]. The functional composition associated with multiple carbon substrate utilization patterns (revealed by EcoPlate) was often linked to species composition [25,34]. Gilbert *et al.* [40] reported a positive correlation between transcript richness (a type of functional richness), and phylogenetic richness by the metatranscriptome approach. These lines of evidence strongly indicated the need to quantify the multifunctionality (MF) of microbial communities. In this way, the role of microbial diversity in ecosystem functioning could be thoroughly evaluated.

A growing social demand exists to better project the future magnitudes of change in decreased microbial diversity, and its consequences on ecosystem functions. Reductions in diversity and shifts in microbial species composition may occur in various ecosystems owing to anthropogenic impacts, e.g. increased nitrogen deposition [41], invasive species introduction and establishment [42], and toxic substance contamination [43]. A quantitative assessment/projection of anthropogenic impacts should be undertaken owing to potential trade-offs between ecosystem functions and services provided from natural microbial assemblages (e.g. regulating services) and ecosystem functions provided from artificial ecosystem modifications (e.g. increased crop production and enhanced bioremediation; [12]). Quantitative MF assessment of natural microbial communities prevents the underestimation of potentially important ecosystem services provided by natural microbial species.

In this study, we provided a new theoretical approach for quantitative evaluation of a microbial community by assessing the following two multifunctional indices: (i) MF and (ii) multifunctional redundancy. As much functional information as possible was incorporated into the evaluation of potential microbial functions by analysing the richness of an evolutionary unit of genetic material, i.e. an orthologue; a gene in different species derived from a common ancestor from speciational processes is an orthologue. Orthologues are generally expected to be functionally conservative; therefore, orthologous genes tend to exhibit a similar function [44]. Because common orthologues are shared by multiple species, we proposed to evaluate orthologue richness in a microbial community, which represents the potential range of functions in the community, and the MF index at the community level. For defining multifunctional redundancy, we used an *orthologue accumulation curve*, which is a graph of the observed orthologue number (i.e. MF) as a function of SR observed in a community. We subsequently hypothesized that the orthologue accumulation curve can be approximated by the power-law relationship, MF = *c*SR^*a*^. The exponent *a* serves as a multifunctional redundancy index, whereas *c* represents the average MF of single species in a community. A smaller *a*-value can be interpreted as larger multifunctional redundancy, indicating that a loss in SR exhibits fewer impacts on MF. These settings are a natural extension defined for redundancy of a single function [21]. This approach provides a new method to quantitatively evaluate the impact of change in microbial diversity on ecosystem functions.

In order to test the above hypotheses, we conducted *community simulations* by integrating genomic and ecological information from the database of microbial metagenomics (microbial genome database (MBGD) for comparative analysis, [45]). We also tested the linkage between MF index defined by the orthologue richness and MF observed in microcosm bacterial communities. This was the initial step to quantify the relationship between microbial diversity and MF of a microbial community. Genomic and ecological information enabled us to conduct extensive simulations, which demonstrated multifunctional redundancy was generally low (0.55 < *a* < 0.75), and therefore quantitatively supported the importance of maintaining microbial diversity.

## 2. Material and methods

### (a) MBGD data compilation

We downloaded the default species set from the orthologue group assignment table (‘default orthologue table’) (‘default’ as of 30 August 2011) from MBGD (http://mbgd.genome.ad.jp/, [45]), which comprised 58 Archaea and 420 Bacteria species; the total orthologue group number was 197 061 (we excluded eukaryotic species and orthologues from the dataset). The single genome can have multiple copies from the identical orthologue group; therefore, we converted the orthologue cluster table into a matrix with binary values (0, 1) by classifying the presence (1) or absence (0) of each orthologue group in each species. The converted table was a simple 478 × 197 061 matrix, which specified the orthologue group present or absent in each species genome. Each of the 478 prokaryotic species in the default species set was derived from different genera (one representative genome from each genus, except for two Archaea species from *Methanococcus*, [45]); therefore, phylogenetic bias was small in the species list. For each species, we first identified genome size, operationally defined as total sequence size of chromosomes + plasmids, based on MGBD data. We also classified each species into 16 habitat, and three oxygen-requirement types (aerobic, anaerobic or facultative) [46,47] (summarized in the electronic supplementary material, table S1). We included orthologue groups with known functions, and groups without specific functional assignments (orthologues predicted as hypothetical proteins or proteins with unknown function, which occupied more than half of the orthologue groups) in the following analyses, to avoid underestimating microbial MF (based on precautionary principle).

### (b) Setting for community simulations

Our objective was to test the power-law relationship between microbial SR, and microbial MF in a community, MF = *c*SR^*a*^, by producing a community orthologue accumulation curve. The list of 478 species was used as the species pool, and we generated several types of ‘pseudo-communities’ from the species pool using four assembly rules. First, we generated domain-specific communities, including Bacterial, Archaeal and prokaryotic communities, which consisted respectively of 420 Bacteria, 58 Archaea and 478 prokaryotes, the latter collectively representing the Bacteria and Archaea taxa. Second, we generated 16 habitat-specific (pseudo-)communities (see the electronic supplementary material, table S1 and appendix S1). Third, we compiled aerobic, anaerobic and facultative communities, based on species oxygen requirements (see the electronic supplementary material, table S1). Forth, with random resamplings from all 478 species, we generated randomly assembled communities with SR from 10 to 470 using an interval of 10. For each SR level, we executed 1000 permutations. The fourth procedure was necessary to assess the influence of an overall community SR on the shape of an orthologue accumulation curve, which acted as a null model.

To evaluate the robustness of the power-law relationship between SR and MF in the pseudo-communities from the default MBGD species pool (which does not ensure the co-occurrence of species in natural environments), five species sets were also prepared: tree holes [16], lake [2], marine [48,49] and our own set from a freshwater pond (see also §2*c*). Each operational taxonomic unit (OTU) was assigned to the most genetically related MBGD strain, and then species sets were constructed for community simulations, which included both of the default and non-default species from MBGD and multiple species from a single genus (see the electronic supplementary material, tables S2–S5 and appendix S1 for detailed methods).

A smooth orthologue accumulation curve was established using the same methods as those applied to generate a species accumulation curve and its rarefaction [50,51]. We estimated the expected orthologue number observed for any smaller number of species in a community under the assumption of random species sampling. The analytical formulae [51] provided the capacity to directly estimate the expected orthologue number, depending on species number. Subsequently, to test the power-law relationship between SR and MF, we used a log–log linear regression of SR versus MF (i.e. ln[MF] = ln*c* + *a*ln[SR]) (see also the electronic supplementary material, appendix S2).

### (c) Microcosm experiment

A microcosm experiment was designed to test the ecological relevance of the orthologue-based approach for quantifying the MF. A water sample was taken from a eutrophicated pond (33.869° N, 132.771° E, Matsuyama, Japan) and spread onto a low-nutrient medium, R2A agar (Becton–Dickinson, Franklin Lakes, NJ, USA) plate. After 5 days incubation at 25°C, a total 60 of colonies were separately picked up and re-streaked onto R2A agar for isolation. Twenty-four isolates were obtained from 60 colonies, and 20 of 24 isolates were used for microcosm experiments as coexistence species in the natural pond water. The selected 20 strains were identified based on 16S rRNA gene sequencing (see the electronic supplementary material, table S6 and appendix S3).

Each isolated strain was pre-cultured for 4 days at 25°C in R2A medium (Wako Pure Chemical Industries Ltd, Japan) and then inoculated into 100 ml R2A medium in 200 ml glass flasks at 1.0 × 10^4^ cells ml^−1^. Inoculation volume of each isolates was adjusted based on their cell abundance determined under a fluorescent microscope [52]. Twenty species microcosm was composed of all the 20 species, whereas 5% reduction of SR was simulated by community microcosms that were composed of 19 species from 20 species (all of 20 combinations [_20_C_19_] were prepared). The experiments were repeated four times on different dates. One experiment composed of the 20 different combination of 19 species microcosms and four to six of 20 species microcosms. Total four replicates of each 19 of 20 species microcosms and 26 replicates of 20 species microcosms were constructed for functional analysis.

We used Biolog EcoPlate (Biolog, Hayward, CA, USA), which is generally used as a measure of substrate usage ability of a bacterial community [25], as the index of community-level MF. An EcoPlate was composed with 31 response wells with different sole carbon sources. The utilization of each carbon source was measured by the colour development of each well (see also the electronic supplementary material, appendix S3). The colour from each carbon source represented the potential functional rate and the pattern of the functional rates from 31 carbon sources represented the multifunctional potential of the assemblage. The MF was measured as the number of substrates that exceeded discrete functional thresholds (*T*). Functional thresholds were set at nine quantiles from 0.1 to 0.9 with an interval of 0.1 based on the minimum and maximum observed functioning (relative absorbance) for each substrate [53,54]. The minimum and maximum were calculated across all experimental compositions in each of four experimental dates because the growth condition was variable depending on experimental dates.

## 3. Results

**Figure 1.**
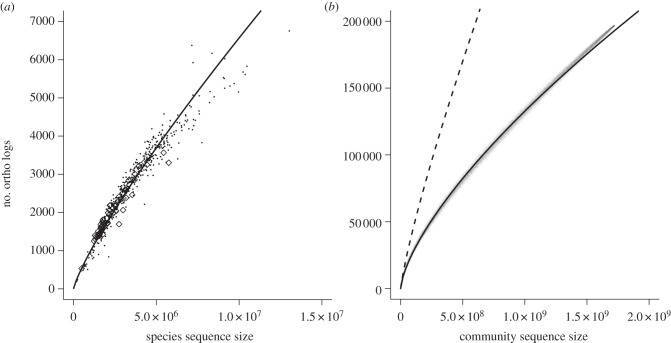
Relationship between genome size and orthologue richness. (*a*) Species sequence size versus orthologue number. Bacteria and Archaea are represented by black dots and open rectangles, respectively. Species regression line is log (orthologue number) = −4.618 + 0.832 × log (species sequence size) (*r^2^* = 0.955, *p* < 0.001). (*b*) Community sequence size versus orthologue number. Community sequence size was calculated for communities generated by resampling methods (see the electronic supplementary material, appendix S2 for more detail) for the orthologue accumulation curve of a prokaryotic community (results are shown as small dots). Community regression line (solid line) is log (orthologue) = −2.475 + 0.689 × log (community sequence size) (*r^2^* = 0.998, *p* < 0.001). The regression line for species is also shown as a dashed line.

We initially detected a positive relationship between each species operational genome size (sequence size) and species orthologue richness for Bacteria and Archaea (figure 1*a*). The positive relationship was maintained between community genome size and community orthologue richness at the prokaryotic community level (figure 1*b*). After we fit the power-law relationship, the species genome slope fit for Bacteria and Archaea species was greater than the fit for the prokaryotic community (figure 1*b*); the exponent *a* expected mean value [95% CI] was 0.8319 [0.8155–0.8482] for the species genome, and 0.6886 [0.6884–0.6889] for the community genome.

**Figure 2.**
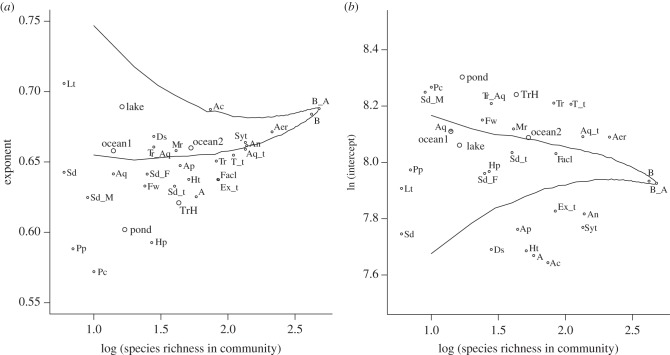
(*a*) Group-dependent exponent and (*b*) intercept from the log–log regression and comparison with randomly assembled communities. OTU classification into groups is shown in the electronic supplementary material, table S1. B, Bacteria; A, Archaea; B_A, B + A; Tr, terrestrial; Aq, aquatic; Tr_Aq, terrestrial and aquatic; Fw, freshwater; Mr, marine; Ds, deep sea; Sd, aquatic sediment; Sd_M, marine sediment; Sd_F, freshwater sediment; Pc, plant commensalism; Ac, animal commensalism; Pp, plant parasitism; Ap, animal parasitism; Ht, high temperature; Lt, low temperature; Hp, halophilic; An, anaerobic; Aer, aerobic; Facl, facultative; T_t, Tr_Aq + Tr; Aq_t, Tr_Aq + Fw + Mr + Aq + Ds; Sd_t, Sd + Sd_M + Sd_F; Syt. Pc + Ac + Pp + Ap; Ex_t, Ht + Lt + Hp. The upper and lower lines represent middle 95% CI ranges from 1000 permutations of randomly assembled communities. TrH, ocean1, ocean2, lake and pond are the results from the microbial communities described in the electronic supplementary material, tables S2–S6, respectively.

Second, we compared the power-law relationships among domain, habitat-type and oxygen-requirement-specific pseudo-communities that were simulated from the default species set in MBGD as well as five communities from natural environments (figure 2). When communities were constructed from the default species set in MBGD, large variation among communities was observed for the exponent (figure 2*a*) and intercept (figure 2*b*). Results from randomly assembled communities indicated that the estimated parameters depended on community SR. The estimated exponents from some communities (e.g. marine (Mr) and deep sea (Ds)) fell into the 95% middle range from 1000 permutations of randomly assembled communities (figure 2*a*), whereas the values from other communities (e.g. sediment community (Sd_t) and extreme community (Ex_t)) deviated from randomly assembled communities. These results indicated multifunctional redundancy can be habitat-dependent. We found similar differences among communities for the estimated intercept that represented the expected MF in a single species (figure 2*b*). For communities that were reconstructed from natural assemblages, the estimated exponents are 0.621 for tree holes (TrH, SR = 43 [16]), 0.602 for the freshwater pond in this study (pond, SR = 17), 0.689 for the freshwater lake (lake, SR = 16 [2]), 0.658 and 0.660 for marine (ocean1, SR = 14 [49] and ocean2, SR = 53 [48], respectively; figure 2*a*), whereas the estimated intercepts are 8.241, 8.303, 8.062, 8.110 and 8.088, respectively (figure 2*b*).

**Figure 3.**
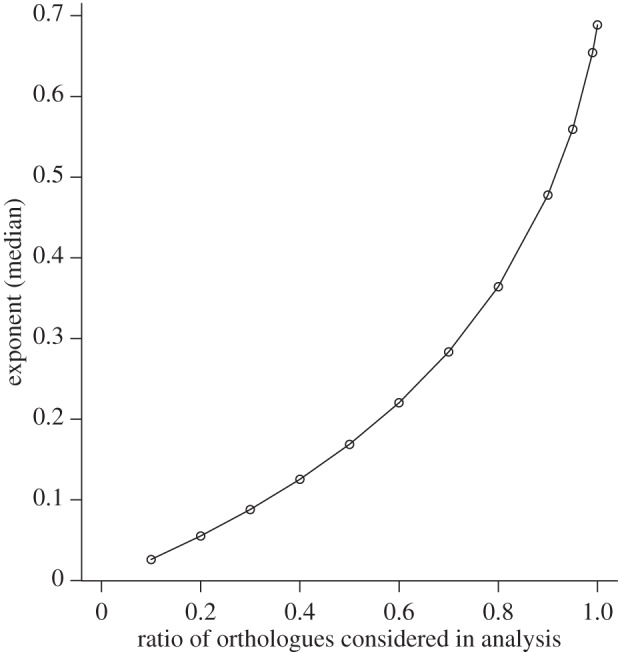
Multifunctional redundancy (exponent *a*) dependence of the prokaryotic community (SR = 478) on the orthologue ratio randomly chosen in simulations from 197 061 orthologues. In 10–99% of cases, the median values from 200 permutations of randomly chosen orthologues are shown. Only median values are indicated because variation among the 200 permutations was very small. The maximum and minimum values among 200 permutations were very close to the median value (i.e. approx. 0.1% from the median).

Next, we investigated the dependence of multifunctional redundancy on the total number of orthologue groups included in the analysis (to estimate the exponent of power function, which is an index of multifunctional redundancy). MF was defined by community orthologue richness under our framework. The orthologue accumulation curve, log–log linear regression fit and the exponent estimate were generated using the reduced number of orthologues; we randomly chose 10–99% of orthologues among 197 061 orthologue groups. The randomization simulation demonstrated that orthologue reduction considered in the orthologue accumulation curve resulted in a substantial decrease in the estimated exponent (the slope of log–log linear regression), indicating higher multifunctional redundancy (figure 3).

**Figure 4.**
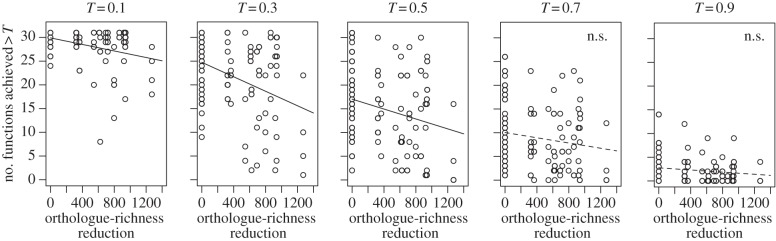
Multifunctionality predicted by orthologue-richness reduction. The effect of orthologue richness reduction on the average number of functions achieved above thresholds (*T*), where *T* is the quantile-based ranking of each function across all treatments in each experiment date. Results where *T* were based on the average functioning were qualitatively similar (see the electronic supplementary material, figure S2). *p*-values from simple linear least-squares regression are < 0.01 (*T* = 0.5), < 0.05 (*T* = 0.6), < 0.001 (*T* = 0.1, 0.2, 0.4), < 10^−4^ (*T* = 0.3), 0.087 (*T* = 0.7), 0.12 (*T* = 0.9) and 0.21 (*T* = 0.8), respectively. n.s., non-significant (*p* > 0.05).

In addition, we investigated whether the MF directly measured in microcosm bacterial communities was predicted by the orthologue richness. The total number of orthologues in the control community that was composed of 20 isolates (which are allocated to 17 distinct strains based on MBGD) was 22 007. The maximum reduction of orthologue in the reduced communities that was composed of 19 isolates was 1272, which is about 5.8% from the control community. The number of functions that exceeded the functional thresholds (*T*), which was the experimental measure of MF, significantly decreased with the reduction of orthologue richness in the experimental assemblages (with *T* = 0.1, 0.2, 0.3, 0.4, 0.5, 0.6; figure 4).

Finally, we proposed a viable means to estimate acceptable levels of a reduction in microbial SR in a community, which did not result in a severe decrease in community MF. Let *x* and *y* denote the reduction (%) in SR, and MF, respectively. From the power-law relationship (MF = *c*SR^*a*^), we easily obtained the following formulae:3.1
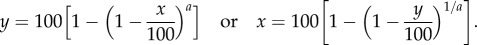


Equation (3.1) shows that more sever reduction (*x*) in SR is acceptable with a larger 1/*a* (i.e. larger multifunctional redundancy) under fixed MF reduction levels (*y*). The exponent (*a*) exhibited large variation among communities (figure 3*a*), therefore a relatively wide range of *a* (0.55 ≤ *a* ≤ 0.75) was used for the following analysis. If we ascertained acceptable MF reduction levels (*y*) based on any ecological or economical conditions and constraints (e.g. less than 1%, 5% or 10%), then we estimated acceptable SR reduction levels (*x*) using equation (3.1) (table 1). For example, if we accepted a 5% reduction in MF (*y* = 5), and adopted the most optimistic case (i.e. the lowest exponent value = the highest multifunctional redundancy: a = 0.55 here) or safest case (i.e. *a* = 0.75), the reduction of SR was less than 8.9% or 6.6%, respectively (table 1). Similarly, if we accepted only a 1% reduction in MF, then the decrease in SR was less than 1.33–1.82%.

**Table 1.** Reduction level estimates for no. sp. (%), which is equivalent to reduction in no. orthologue (*y*) 1%, 5% and 10%. (Exponent (*a*) values cover a broader range than those shown in the figure 2 for precautionary purposes. Note that most exponent values shown in figure 2 are greater than 0.60.)

reduction of no. orthologue (%)	reduction of no. sp. (%) exponent *a*				
	0.55	0.60	0.65	0.70	0.75
1	1.8174	1.6611	1.53431	1.4255	1.33111
5	8.90439	8.19366	7.58795	7.06558	6.61048
10	17.4334	16.1047	14.9638	13.9735	13.106

## 4. Discussion

### (a) The relationship between biodiversity and multifunctionality

The importance of SR for maintaining ecosystem MF has been demonstrated in plant [39,55] and microbial communities [27,39,56]. In this study, we proposed a new method for quantifying MF in a microbial community, which uses accumulating metagenomic information. We defined orthologue richness in the community as MF. We also defined multifunctional redundancy by the exponent of power function estimated by fitting the power function to the orthologue accumulation curve; the larger the exponent, the lower the multifunctional redundancy. Our community simulations demonstrated that multifunctional redundancy is generally low. Reich *et al.* [21] showed that monofunctional redundancy became lower through time using two datasets derived from grassland biodiversity experiments; the largest exponent values were 0.42 and 0.51 in each dataset. Our simulations generated exponents ranging from 0.57 to 0.71 (figure 2*a*), which suggests lower multifunctional redundancy rather than monofunctional redundancy, as reported in Gamfeldt *et al.* [39]. Deep sea benthic metazoan communities also show low multifunctional redundancy; the functional diversity (trait diversity) is the power function of SR with the exponent 0.59 [57]. These results imply the consistency of limited multifunctional redundancy in terrestrial plant, metazoan and prokaryotic communities.

In our study, the estimated exponent (0.832), which was less than unity, derived from the relationship between species genome size and orthologue richness (figure 1*a*) suggested a certain degree of multifunctional redundancy within an individual genome. It might be argued that orthologue richness in a community can be predicted (extrapolated) by community genome size using the power-law relationship fit within a genome (figure 1*a*). However, community simulations demonstrated that orthologue richness in a community was much smaller than predicted by the relationship within a genome (figure 1*b*). In addition, estimated multifunctional redundancy was higher (i.e. estimated exponent 0.689 was lower) than within an individual genome (exponent 0.832). These results indicated the community cannot be regarded as a single *super*species with a very large genome. Instead, sharing common orthologues among species is a key mechanism, responsible for multifunctional redundancy (figures 1*b*, 2 and 3).

Randomization simulations indicated that multifunctional redundancy (exponent *a*) was influenced by the degree of data coverage: SR (figure 2*a*) and orthologue richness (figure 3). However, concurrently multifunctional redundancy differed among domains, habitats and oxygen requirements (figure 2*a*), suggesting multifunctional redundancy was dependent on habitats and environmental conditions in natural systems. Furthermore, when the estimated exponent in a specific community was outside the 95% CI from randomly assembled communities, the exponent was mostly less than the lower 95% CI limit (figure 2*a*). These results indicated that genome orthologue composition similarities among species in a specific community can be significantly higher than in randomly assembled communities, suggesting environmental selection (environmental filtering [11]) on orthologue composition. Between domains, multifunctional redundancy was higher in Archaea (A, SR = 58) than Bacteria (B, SR = 420). When the redundancy was separately calculated for each domain in extreme environment community (Ex_t), the exponents were 0.589 and 0.625 for Archaea (SR = 30) and Bacteria (SR = 54), respectively, indicating the higher redundancy in Archaea. It might imply the stronger environmental selection on Archaea community and/or phylogenetically more aggregated choice of species from MBGD for Archaea. Between habitats, an interesting pattern from pseudo-communities is that the marine environment (Mr) showed lower redundancy than the freshwater environment (Fw). Between oxygen requirements, it is worth noting that the facultative groups (Facl) had higher redundancy than anaerobic (An) and aerobic (Aer) groups. Although there are only five examples, results from natural assemblages (TrH, pond, lake, ocean1, ocean2) may imply that the synthesized communities of isolated strains from the identical media (pond and TrH) show functionally more redundant than communities described by non-cultured methods (lake and ocean1, ocean2; the exponent from TrH and pond are much smaller than lake and oceans).

### (b) Theoretical implications of community simulations and future directions

Many gene functions remain elusive despite sequence analyses. For example, in the MBGD database, more than 180 000 orthologues are classified simply as hypothetical proteins. Characterizing the physiological traits of prokaryotes is challenging, even for isolated species. Orthologues (genes that presumably share functions) are an effective unit of distinct function, and a cautious approach to avoid underestimating microbial functions, assuming higher orthologue richness implies higher functional diversity or MF. Our simulations found that removing orthologous genome segments from the analysis overestimated multifunctional redundancy (figure 3). In general, species functions are linked to *ecosystem* functions, which are characterized by functional traits. These traits determine how organisms respond to environmental changes or effect the environment [11]. *Ecological* functions are characterized by ecological traits, which are responsible for ecological interactions between the focal species and other species. Therefore, it is reasonable to assume that all functional genes are potentially related to ecosystem and/or ecological functions, which, in turn, participate in the diverse array of ecosystem services [12].

Orthologous genes in different species have diverged from a single gene in a common ancestor. Therefore, increased richness of orthologue groups in a community suggests the maintenance of diversification processes during prokaryotic evolution. In other words, MF, which is defined as community orthologue richness, can be characterized as functional diversity, but also evolutionary diversity. Therefore, our approach provides a means to incorporate an evolutionary perspective into biodiversity science (cf. [58] a phylogenetic diversity measure [59]).

Owing to the limited availability of ecological information in genome databases (e.g. only 16 habitat types) [46,47], MBGD pseudo-communities may include species that do not co-occur in natural environments. Therefore, additional examples of a natural microbial community are valuable to evaluate the robustness of the patterns discovered from MBGD pseudo-communities. These data will provide the concrete SR–MF relationship in a specific environment as is shown in our five examples (figure 2). Multifunctional redundancy comparisons between/among different environments generate a more robust depiction of regional variation in the vulnerability of a microbial community. In addition, species versus orthologue richness can be plotted using data from many regions, which provides biodiversity–MF relationships at larger spatial scales. From methodological point of view, it is also notable that the similarity of naturally occurring species found by a culture-independent method to the most genetically related strain in a genome database can be lower (e.g. lower than 90%; see the electronic supplementary material, table S4) than species found by a culture-dependent method (greater than 97%; see the electronic supplementary material, table S6), owing to the dominance of unculturable species in natural assemblages. Under the limited availability of information of uncultured species in a genome database, the combination of culture-independent and culture-dependent approaches would lead to better understanding of the link between microbial SR and genetic (orthologue) richness in natural environments.

Our microcosm experiment was the first step to ascertain the relationship between orthologue richness and microbial processes. Although the degree of manipulation of SR and orthologue richness was small (about 5% reduction from the control), we found that the reduction of the MF directly measured by carbon substrates usage ability could be predicted by the reduction of orthologue richness (figure 4). This result strongly implied the link from potential MF to the actual expression of multiple functions. At the same time, the predictability was generally low (e.g. adjusted *r*^2^ = 0.14 when *T* = 0.3 in figure 4) probably, because we count all of the orthologues equally independently of whether or not their functions are already predicted in MBGD. More detailed analysis on genes with ecological functions will improve the reliability of MF index predicted from genomic data. More interestingly, the low predictability would imply that the community-level functions cannot be fully predicted just by the sum of genetic functions of each species; inter-specific ecological interactions might also matter. Larger scale experiments with larger variations of microbial SR and orthologue richness than our settings will also elucidate the effectiveness of community orthologue richness as the index of ecosystem processes and MF.

### (c) Importance of quantitative information for conservation and sustainable biodiversity use

Former studies that focus on a single ecosystem function or service (e.g. productivity, nutrient retention and resistance to species invasion) indicated that direct supporting evidence of the importance of species number was limited by the small number of species present (10 or fewer) [60]. Therefore, Diaz *et al.* [60] reported the possibility that ‘a reduction in the number of species may initially have small effects’ was difficult to exclude. However, applying a new community MF measure (using a prokaryotic community as a model), our theoretical result demonstrated that even an initial small loss of SR had proportional effects on community (multi)functionality (equation (3.1) and table 1) as well as our experimental result from the microcosms (figure 4). In other words, the levels of reduction in SR required to avoid substantial declines in ecosystem services must be very low (e.g. less than 10%). It is also worth noting that our simulations confirmed a general hypothesis generated in other studies that multifunctional redundancy increases as functional diversity decreases (figure 3) [27,39,55,56].

A contemporary concern for biodiversity conservation is that decision makers require quantitative biodiversity evaluations as part of science-based negotiations and communications. The Convention on Biological Diversity (CBD), Article 14 addresses the importance of appropriate assessment to minimize adverse effects of anthropogenic impacts on biological diversity [61]. For example, the introduction of living modified organisms (LMOs), including crops and microorganisms to natural environments is suspected to result in unfavourable changes to microbial communities supported by soils and watersheds, leading to a loss in microbial diversity and ecosystem function. The Cartagena Protocol on biosafety (a supplement to CBD) requests decision-making based on scientifically sound risk assessments to identify and evaluate the potential adverse effects of LMOs on the conservation and sustainable use of biological diversity [62]. However, the quantitative methods to assess the relationship between biological diversity and ecosystem MF remain underexplored, which prevents any quantitative assessment of LMOs adverse effects, leading to biological diversity and ecosystem services decline. This is the case not only for LMOs. In general, enhancement of one ecosystem service in agro- (such as crop production) and natural ecosystems is accompanied by changes in community structure and SR, which in turn degrades other ecosystem services (e.g. water purification) [12]. Under such trade-offs among different ecosystem service components, and increasing public demands for sustainable biodiversity use that balances costs and benefits, quantitative evaluation of the impacts of biodiversity loss on MF will have greater future importance. The approach proposed in this study serves as a foundation for additional risk assessment developmental procedures to facilitate scientifically sound international decision-making.
